# Prediction of Bolt Loosening Life: A Practical Approach Considering Variable Amplitude Loading and Multi-Bolted Structures

**DOI:** 10.3390/ma18051069

**Published:** 2025-02-27

**Authors:** Min Yang, Seong-Mo Jeong, Seong-Gu Hong, Jae-Yong Lim

**Affiliations:** 1 Department of Safety Engineering, Seoul National University of Science and Technology, Seoul 01811, Republic of Korea; ym20324011@gmail.com (M.Y.); smcrom@seoultech.ac.kr (S.-M.J.); 2 Convergence Research Center for Meta-Touch, Korea Research Institute of Standards and Science, Daejeon 34113, Republic of Korea; 3 Division of Chemical and Material Metrology, Korea Research Institute of Standards and Science, Daejeon 34113, Republic of Korea; 4 Department of Applied Measurement Science, University of Science and Technology, Daejeon 34113, Republic of Korea

**Keywords:** bolt loosening, bolt loosening life, multi-bolted structures, damage accumulation, two-block loading

## Abstract

Bolted connections are crucial in joining mechanical assemblies and ensuring the integrity and reliability of structural components. This study proposes a method for estimating bolt loosening life by practically applying material fatigue life prediction methods. First, the study employed the linear cumulative damage rule to predict the loosening life of single bolts under two-block loading conditions. Second, a test device with two bolt attachment points on a single structure was fabricated to model the multi-bolted structure, and tests were conducted. Finite element analysis (FEA) analysis was employed to identify vulnerable points. The loosening life of single bolts predicted using the linear cumulative damage rule exhibited enhanced accuracy within a ±1.2× error band compared with the experimental data despite variations in bolt types and test conditions. The FEA results for the multi-bolt structure demonstrated that the loosening life could be predicted by identifying vulnerable points and estimating the displacements. This study effectively predicts the bolt loosening life, offering valuable data for the reliability assessment of bolted structures.

## 1. Introduction

Bolted connections are crucial in joining mechanical assemblies and ensuring the integrity and reliability of structural components. They are widely utilized owing to their advantages, such as ease of assembly, versatility, load distribution, cost-effectiveness, and accessibility for maintenance. However, when the bolts are subjected to vibration, shock, or cyclic loading, the original force equilibrium on a threaded surface can be disrupted, leading to relative slippage and rotation between the bolt and nut [1,2]. This can decrease the initial clamping force, leading to catastrophic failure [3,4,5].

Numerous studies have been conducted to examine bolt-loosening mechanisms and effective prevention methods [6,7,8]. Pai and Hess [9,10] first identified that fasteners can loosen under dynamic shear loading due to localized slip accumulation. Building on this, Izumi [11] and Dinger [12] further argued that localized slip leads to the gradual loosening of bolted joints. Yang et al. [13] observed that bolt loosening generally precedes fatigue fracture, whereas, under high axial loads, bolts may fail due to fatigue without significant loosening. Jiang et al. [14] demonstrated that bolt loosening under transverse cyclic loading follows a pattern similar to fatigue curves with an endurance limit. Yang et al. [15] further analyzed the relationship between displacement amplitude and bolt loosening life, revealing similarities to material fatigue S–N curves. Fan [16] and Zhang [17] demonstrated that thread fretting wear under transverse cyclic loading leads to a gradual reduction in clamping force in bolted joints. Liu et al. [18] studied coated bolts, showing they reduce thread damage and improve clamping force retention, enhancing self-loosening resistance. However, most studies have primarily focused on the bolt-loosening mechanism and have been limited to single-bolt joints.

In real-world applications, bolted structures experience a complicated load history rather than sinusoidal vibration loading, making laboratory-based findings difficult to apply directly. However, studies on bolt loosening life under realistic service conditions remain limited. To effectively address that issue, the vulnerability of bolt loosening in real-life structures under variable amplitude loading must be identified to predict the bolt loosening life in those identified vulnerable areas. Because bolt loosening life is a crucial indicator for evaluating the structural capacity of bolted connections, bolt loosening life assessment verification is essential [19,20].

This study aims to demonstrate the practical application of fatigue life prediction methods for bolt loosening life prediction. The first is the prediction of bolt loosening life under variable amplitude loading (VAL) using the linear cumulative damage rule (LDR). The second is the prediction of bolt loosening lives in a multi-bolted structure simulating real structures. Finite element analysis (FEA) was employed to identify areas vulnerable to bolt loosening in multi-bolted structures. Subsequently, validation tests were conducted to confirm the bolt loosening life in structures with multiple bolt connections based on the FEA results.

## 2. Materials and Methods

### 2.1. Test Methods for Bolt Loosening Test of Two-Block Loading

**Figure 1:**
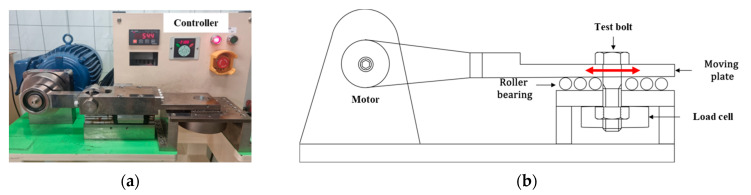
Junker tester: (**a**) test machine and (**b**) schematic. (Reprinted with permission from International Journal of Precision Engineering and Manufacturing) [21].

Two types of bolts (M6 × 1.0 × 65 and M8 × 1.25 × 65) were used for the variable-amplitude loading tests, as in our previous study [21]. Bolt loosening life tests of the two-block loading were conducted using a Junker tester, as shown in Figure 1. Cumulative fatigue damage theory [22,23,24,25] was applied to predict bolt loosening life, where the bolt loosening damage *D* by constant amplitude loading after $n_{i}$ cycles can be expressed as follows:

(1) $$D=\sum \frac{n_{i}}{N_{i}}$$

where $n_{i}$ denotes the number of constant amplitude loading cycles at *i*-th displacement amplitude, and $N_{i}$ is the bolt loosening life for the corresponding constant amplitude load. If the linear LDR is used for loosening life prediction, the bolt must be loosened when damage *D* reaches unity. The initial clamping force, frequency, and sampling rate were identical to those used in a previous study, as shown in Table 1 [21].

**Figure 2:**
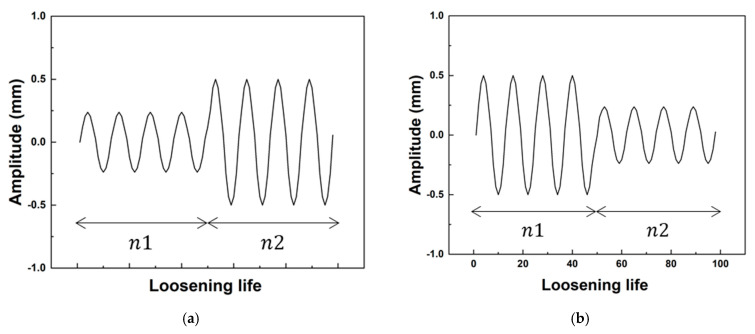
Two-block loadings: (**a**) Lo-Hi and (**b**) Hi-Lo.

The two-block loading tests were designed based on previous constant amplitude bolt loosening tests [21]. As shown in Figure 2, the applied load sequence consisted of an initial displacement-controlled cycle, utilizing 50% of the expected bolt loosening life at the given displacement amplitude, followed by additional cyclic loading at a different amplitude until loosening occurred. For example, in Test 1, a displacement amplitude of 0.3 mm was applied for 12,000 cycles, followed by cycles at 0.65 mm until loosening. A similar procedure was followed for other test conditions. Table 2 illustrates the detailed procedures for the tests, including the bolt size, two-block loading, and number of tests.

### 2.2. Prediction of Bolt Loosening Life for Multi-Bolted Structures

This section discusses the finite element (FE) model construction and testing method for bolt loosening life prediction of multi-bolted structures. The geometry of the FE model was constructed to simulate multi-bolted structures. To verify the FE results, a test fixture was fabricated using the FE model.

#### 2.2.1. FE Model Construction for the Multi-Bolted Structure

Three-dimensional FE models were constructed to identify the most vulnerable regions in the bolt structure regarding the displacement amplitudes. Accurately determining the relative displacement at the bolt connection is crucial for predicting the bolt loosening life.

**Figure 3:**
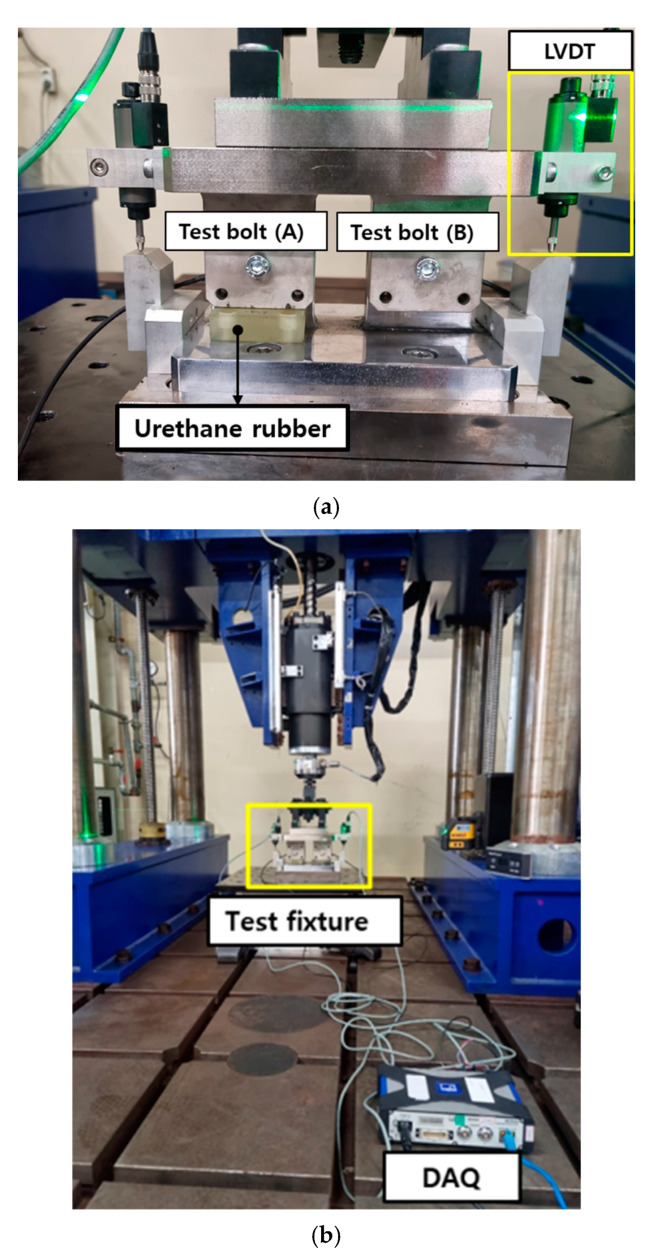
Test device installed on the vibration durability test machine. (**a**) test fixture (**b**) test setup.

An FE model was constructed based on the test fixture (Figure 3a). In the FE model, 8-noded 3D solid elements (13,798 elements) with reduced integration and 6-noded 3D solid elements (6-node linear triangular prism elements) for the plates in the bolt structure were used. The material of the plates was SCM440, modeled using an elastic-plastic hardening model [26,27] with an elastic modulus of 200 GPa and a Poisson’s ratio of 0.3. A bolt connection element was used as the connector element.

To optimize computational efficiency, a simplified single equivalent element model (CONN3D2 in ABAQUS 2023 notation) was employed instead of a detailed FE model of the bolt geometry. This equivalent element integrates an isotropic hardening plasticity model to capture the bolt’s cyclic behavior while significantly reducing the computational cost.

At the bolt connection, when the bolt is subjected to transverse cyclic loading, bending moments occur under the bolt head and on the threaded surface, which is crucial for analyzing the mechanical behavior of the bolt. When applying this theory to a cantilever subjected to bending loads, the Timoshenko beam theory [28,29,30], which accounts for shear deformation, was implemented in the FEA model.

The transverse displacement, *ω*(*x*), against transverse load, *P*, is determined as follows:

(2) $$\omega \left(x\right)=\frac{P(L-x)}{kAG}-\frac{Px}{2EI}\left(L^{2}-\frac{x^{2}}{3}\right)+\frac{PL^{3}}{3EI}$$

At *x* = *L*, the Equation (2) becomes the following:

(3) $$\omega \left(L\right)=P(\frac{L}{kAG}+\frac{L^{3}}{3EI})$$

The stiffness in each Cartesian direction was calculated based on the applied force and resulting displacement. The stiffness values $k_{x}$ and $k_{y}$ of the bolt can be expressed using the following relationships:

(4) $$k_{x}=\frac{P_{x}}{\omega (L)}=\frac{1}{\frac{L}{kAG}+\frac{L^{3}}{3EI}}$$

(5) $$k_{y}=\frac{P_{y}}{\omega (L)}=\frac{1}{\frac{L}{kAG}+\frac{L^{3}}{3EI}}$$

where *L* is the bolt length, *A* is the effective cross-sectional area of the bolt, *E* is the elastic modulus of the bolt, *I* is the second moment of the cross-sectional area, and *G* is the shear modulus of the bolt. *κ* is the Timoshenko shear coefficient, which varies depending on the shape, and in this study, Equation (3) was applied for the circular cross-sectional shape of the bolt.

(6) $$\kappa =\frac{6(1+v)}{7+6v}$$

where v is the Poisson’s ratio. The following is for a structural steel with v = 0.3:

κ=0.886

Stiffness $k_{z}$ in the axial direction of the bolt can be calculated using the following equation:

(7) $$k_{z}=\frac{AE}{L}$$

Table 3 illustrates the stiffness values calculated in the $k_{x}$, $k_{y}$, and $k_{z}$ directions using Equations (4), (5), and (7), respectively. Moreover, the bolt is represented as a connector element, essential for accurately capturing the mechanical behavior of the bolted connection. The analysis was conducted using the specific stiffness values defined for the connector element.

**Figure 4:**
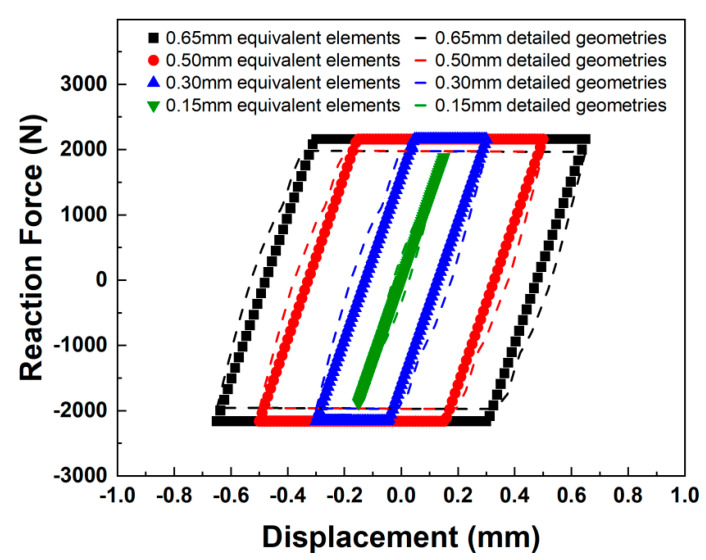
Comparison of the mechanical behavior of bolts of equivalent elements and detailed geometry models: M8 bolt.

Figure 4 compares the cyclic behavior obtained from the FEA using the detailed FE model and that obtained from the analysis using a single equivalent element. The hysteresis curves of the M8 bolts were compared, with each color representing a different displacement amplitude. The dotted lines represent the hysteresis curves between the reaction force and displacement obtained in a previous study [21], and the symbols represent the equivalent element calculations of these curves. However, no slip occurred under a displacement amplitude of 0.15 mm. Moreover, the hysteresis loops expanded with increasing transverse displacement. Under the conditions that exceeded the 0.15 mm displacement amplitude, complete slip occurred in the zero-slope regions, leading to the reaction forces of the initial clamping force of the M8 bolt (14.3 kN) multiplied by the friction coefficient (0.15) specified in the FE model.

As the difference in the reaction forces between the two FE models of the hysteresis curves is insignificant, the description of the cyclic behavior using the equivalent bolt element was successful. The two FE models showed 7.1% and 8.0% differences in stiffness and peak reaction force, respectively. In addition, its computational efficiency is obviously beneficial, especially for the FE analysis of structures having a lot of bolt connections.

Because the test fixture in Figure 3a was designed to provide additional stiffness on one bolt-jointed side, a pad made of urethane rubber (15 t), natural rubber (15 t), or a combination of steel/urethane (12 t/3 t) was placed. The mechanical properties of each rubber material were obtained from additional compression tests, as specified in the ASTM D575 standard [31]. The neo-Hookean model was employed to model the mechanical behavior of the rubber material [32]:

(8) $$W=C_{1}(I_{1}-3)$$

where W is the strain energy function, $C_{1}$ is the nonlinear material constant determined by the stress–strain behavior obtained from the rubber material tests, and $I_{1}$ is the principal strain invariant.

In the static analysis, all the degrees of freedom on the top and bottom surfaces of the text fixture were restrained except in the vertical direction of the top surface. A vertical displacement of 1 mm was applied to the top surface of the text fixtures.

#### 2.2.2. Test Methods for Multi-Bolted Structures

To simulate the multi-bolted structures, a test fixture was fabricated with two bolt attachment points on a single structure to generate different displacement amplitudes, as shown in Figure 3a. Figure 3a illustrates that one of the two bolt attachment points (TEST bolt (A)) was fixed to the bottom plate using urethane rubber to induce a physically smaller displacement than the other attachment points (TEST bolt (B)). Two parts made of SCM440 steel were designed to enhance the mechanical durability of specimens subjected to Q/T (tempering after quenching). Two parts with 10 mm diameter holes were used for M8 bolt tests and transverse displacement movement.

**Figure 5:**
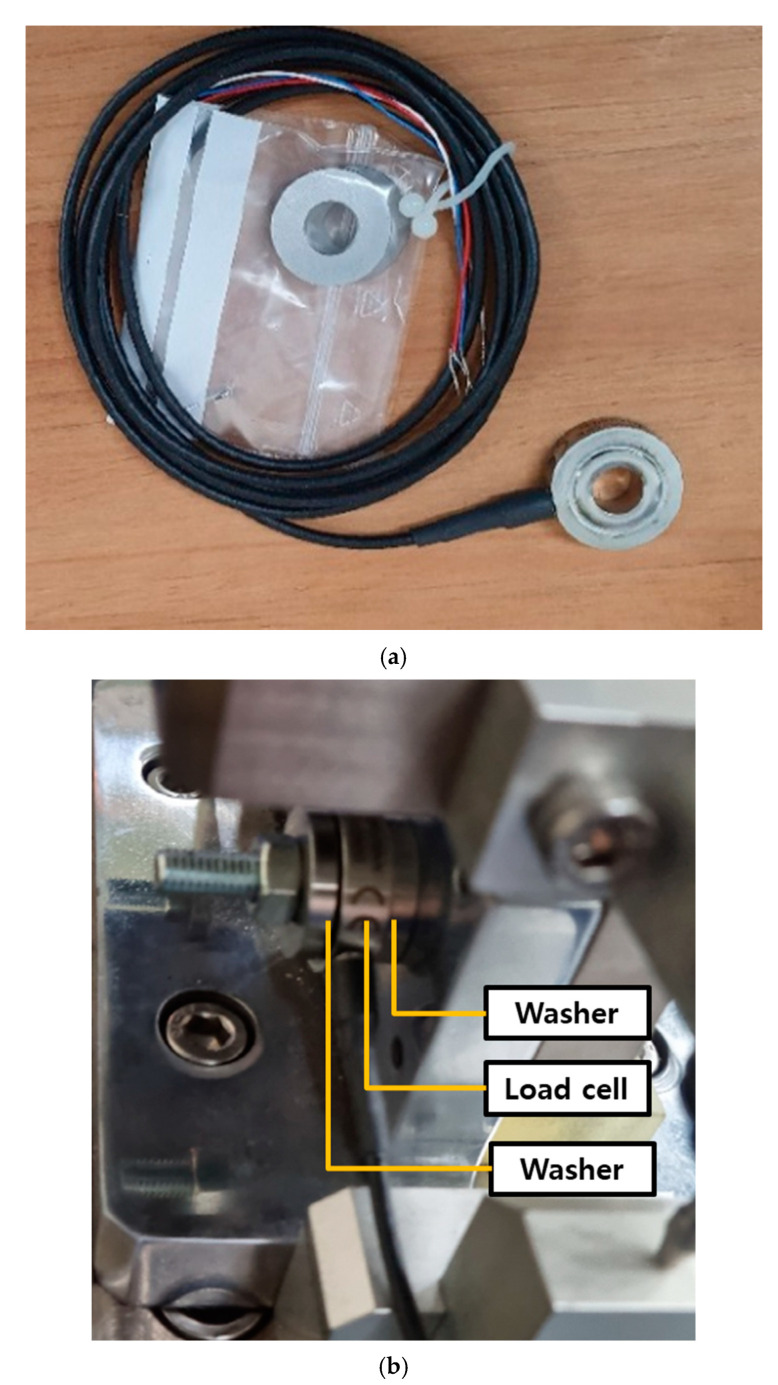
Sensor for measuring bolt clamping force: (**a**) washer-type load cell and (**b**) two washers.

The multi-bolt structure tests were conducted by assembling the test fixture manufactured on the vibration durability equipment, as shown in Figure 3b. The test bolt used was M8 × 1.25 × 65, and the initial clamping force was set to 14.3 kN using a torque wrench. A washer-type load cell (HBMKMR-60 kN, Darmstadt, Germany) was used to measure the bolt clamping force, as shown in Figure 5a. A washer-type load cell was inserted between the two washers to achieve real-time and uniform measurement of the bolt clamping force during the bolt loosening experiments, as shown in Figure 5b. Contact-type linear variable displacement transducer (LVDT) sensors were used to measure the transverse relative displacement of the test bolts, as shown in Figure 3a. All data were recorded using a DAQ system (HBM^®^ QuantumX MX840, Darmstadt, Germany).

Bolt loosening life tests for multi-bolted structures were performed under various conditions, as illustrated in Table 4, with displacement amplitudes of 0.5 mm, 0.4 mm, and 0.3 mm. Each experiment was repeated twice. The tests were concluded when the bolt fractured or its clamping force reached the point whereby the initial preload decreased by 20%, whichever occurred first.

## 3. Results and Discussion

### 3.1. Results of the Bolt Loosening Life Prediction Under Two-Block Loading

This section discusses the results of the life prediction of bolt loosening under two-block loading, demonstrating the feasibility of applying the linear LDR to predict bolt-loosening lives. The experimental life refers to the life obtained from the tests. However, the predicted life is calculated based on the LDR and D-N curve [21], as discussed in Section 2.1. This defines the relationship between the displacement amplitude and bolt loosening life obtained through experiments by applying a constant displacement amplitude to individual bolts in previous studies. This D-N curve can be considered a characteristic curve for individual bolts, analogous to the material properties.

**Figure 6:**
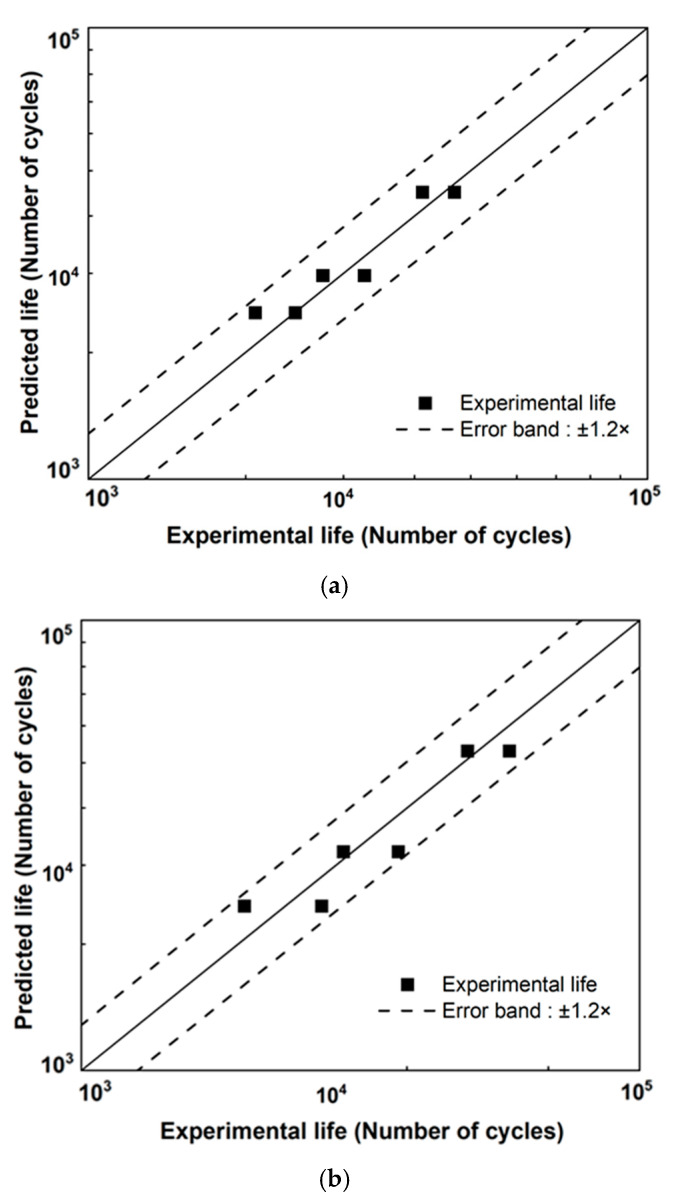
Comparisons between experimental and calculated loosening predicted life using the linear damage rule: (**a**) M8 and (**b**) M6 bolts.

Figure 6a,b compare the test lives and predicted ones for M8 bolts and M6, respectively. The abscissa and ordinate represent the bolt loosening life under the two-block loading and predicted bolt loosening life based on the bolt loosening properties (D-N curve) [21], respectively. The black dashed lines represent the upper and lower bounds within the ±1.2× error band.

**Figure 7:**
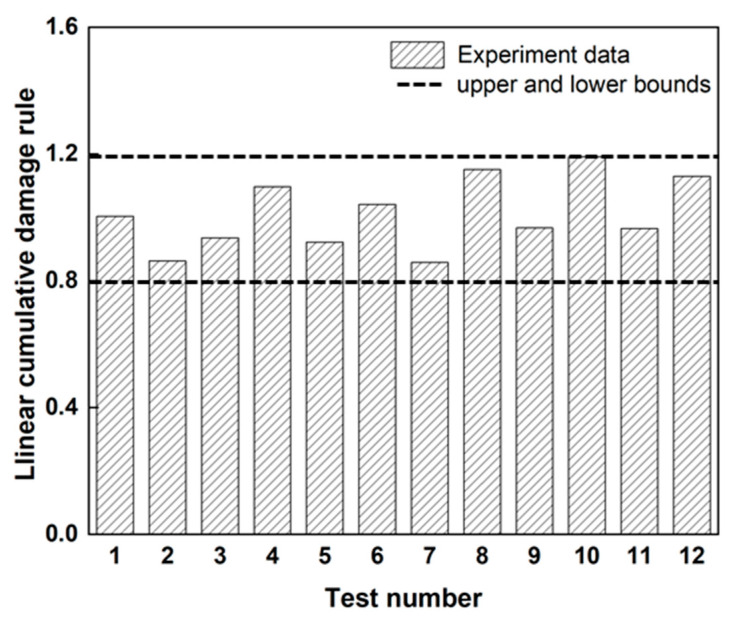
Application of linear cumulative damage rule using VAL test results of M8 and M6 bolts.

Figure 7 shows all the loosening life test lives under the two-block loading for the M8 and M6 bolts. Damage D in linear LDR [22] ranged from 0.8 to 1.2, as determined by the VAL test results and expressed by Equation (1). These findings confirm that the proposed approach provides a reliable and accurate prediction of bolt loosening lives under variable amplitude loading (VAL). Consequently, bolt loosening life prediction using LDR provides excellent results that show promise as a valuable prediction method for this VAL. However, as bolt loosening can be influenced by various factors such as temperature fluctuations, lubrication, and corrosion, the applicability may vary, or perhaps new D-N curves considering those may be required. Additionally, accuracy can be affected if the displacement amplitude becomes larger than considered here or the loading sequence effect becomes significant.

### 3.2. Prediction of Bolt Loosening Life of Multi-Bolted Structures

This section discusses the expected bolt loosening life of multi-bolted structures from two perspectives. First, FEA was employed to identify the regions susceptible to bolt loosening. Next, we compared the predicted bolt loosening life with the experimentally measured loosening life using a previously established D-N curve, which indicates the relationship between the displacement amplitude and bolt loosening life. This curve was determined through experiments in previous studies characterizing loosening behavior.

#### 3.2.1. Identification of Vulnerable Regions Using FEA

**Figure 8:**
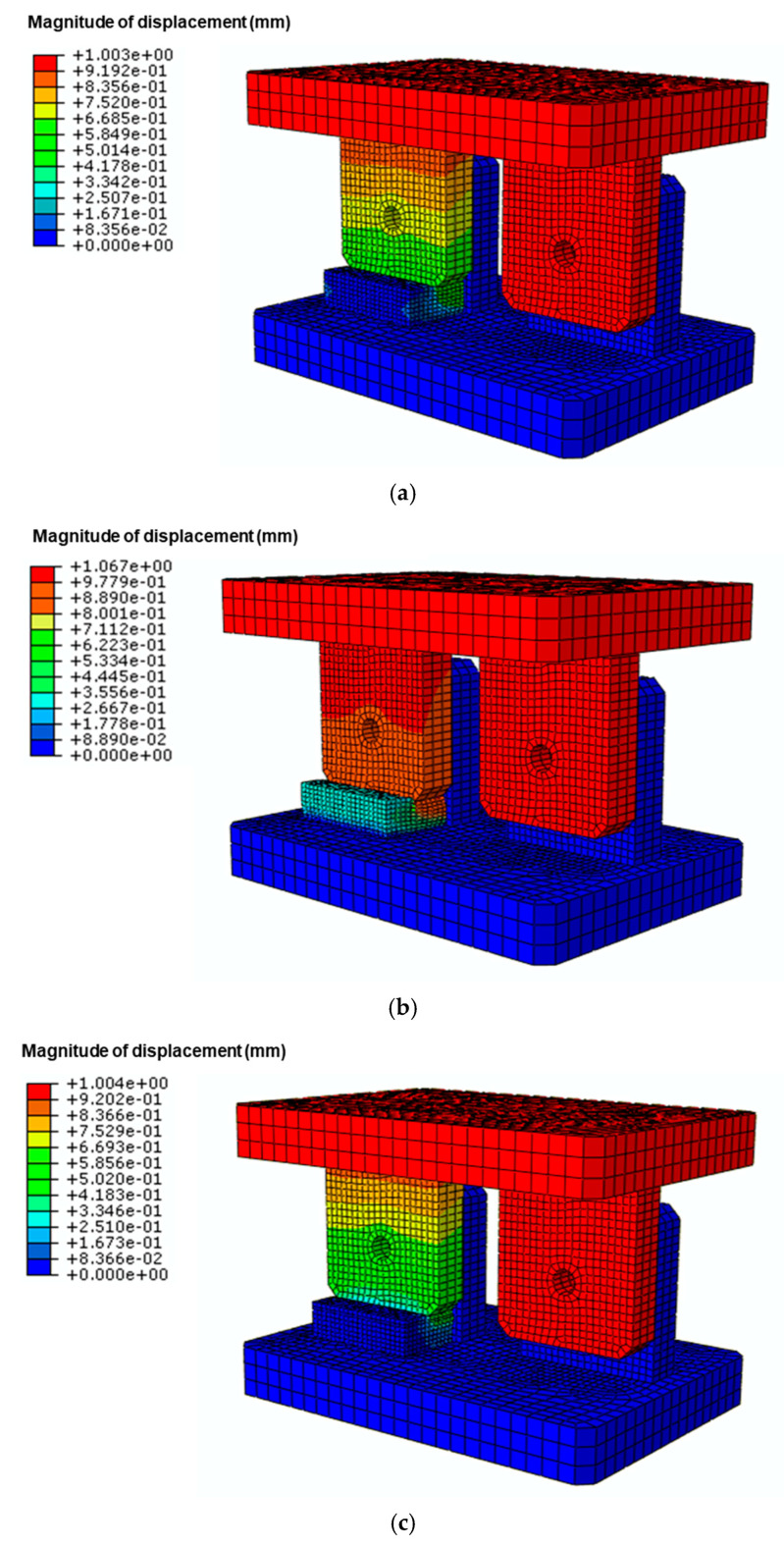
Contour of the magnitude of displacement when one of the following is inserted between the upper and lower parts: (**a**) urethane rubber (15 t), (**b**) natural rubber (15 t), and (**c**) combination of urethane/steel plate (3 t/12 t).

Figure 8 illustrates the simulation results for the relative displacement amplitudes of the multi-bolted structures. When a vertical displacement of 1.0 mm was applied downwards, the bolt connection without pads experienced a physical displacement amplitude of Δd = ±1.0 mm. However, the displacement amplitudes of the other connections were Δd = 0.7 mm, 0.96 mm, and 0.55 mm when urethane rubber (15 t), natural rubber (15 t), and a combination of steel plate/urethane (12 t/3 t) were placed, respectively. A point with a larger relative displacement in the bolt connection was more vulnerable to loosening, indicating that the bolt in the connection loosened first. Thus, bolt connections without pads would be more susceptible to loosening because the displacement amplitude at the connection was larger.

In summary, FEA is an effective tool for identifying loosely vulnerable regions in engineering structures under different displacement amplitudes. By combining this with the individual bolt loosening life database (i.e., D-N curves), the first loosening site and its corresponding loosening life can be estimated because FEA provides the largest displacement amplitude and location information.

#### 3.2.2. Estimation of Bolt Loosening Life in a Multi-Bolted Structure

This subsection discusses the predicted loosening life at vulnerable connections identified using FEA and compares them with the experimental results. Table 5 lists the relative displacement values of each bolt connection when the displacement amplitude conditions for the three cases in Table 4 were applied using the vibration durability equipment (Figure 3b). In the multi-bolted structure experiment, test bolt (B) without the pad exhibited a larger displacement than test bolt (A), consistent with the FEA results, as illustrated in Table 5. Test bolt (B) without the pad nearly matched the amplitude displacement values applied by the durability equipment. However, the test bolt (A) with the pad exhibited a relatively smaller displacement amplitude (±0.2 to ±0.25 mm) compared to the test bolt (B). Thus, the test bolt without pad (B) loosened first, owing to its larger relative displacement, making it more susceptible to loosening.

**Figure 9:**
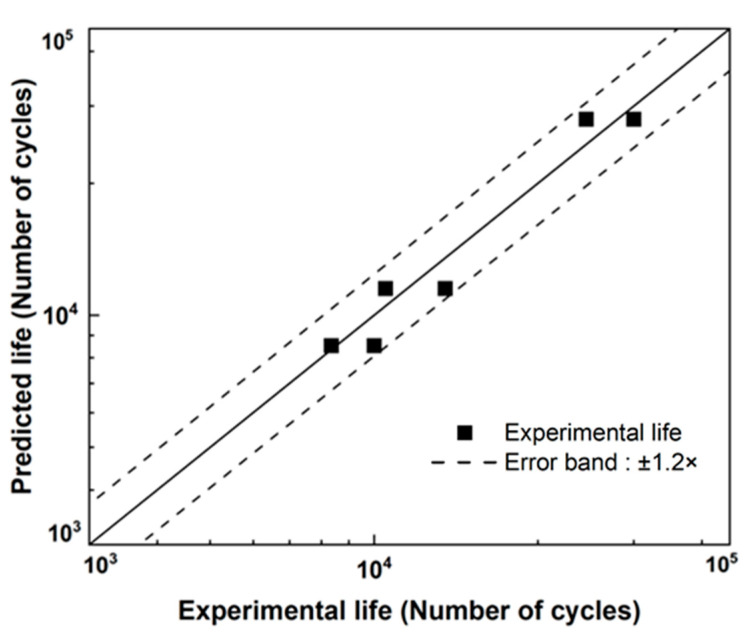
Comparison of real structure bolt loosening life test and loosening life prediction.

**Figure 10:**
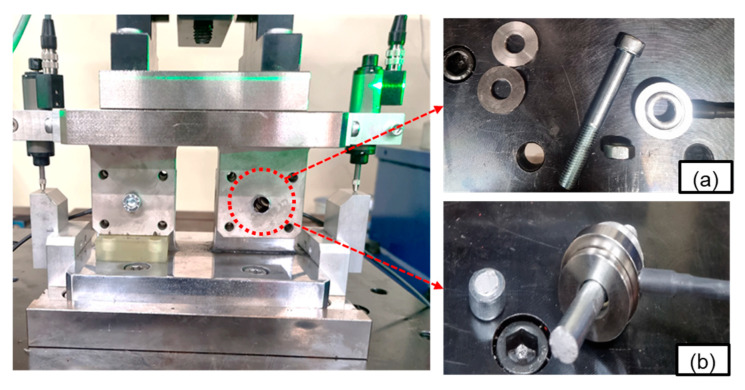
Bolt loosening phenomenon examination: (**a**) bolt loosening (Δd = ±0.3 mm) and (**b**) fatigue fracture (Δd = ±0.5 mm).

We conducted the tests under high-cycle loosening conditions ($10^{4}$ and $10^{5}$ cycles) because small amplitude displacements contribute to bolt loosening. The bolt loosening life fell within the range of approximately $10^{4}$ and $10^{5}$ cycles in all tests, as shown in Figure 9. In Cases 1 and 2, the clamping force of test bolt B initially decreased by approximately 10% at a constant rate, followed by a more rapid decrease. Subsequently, bolt loosening was observable, as shown in Figure 10a. In Case 3 (Δd = ±0.5 mm), the clamping force decreased by 5–7% during the first approximately 100 cycles, followed by a stable decline before sharply dropping again, resulting in bolt fracture, as illustrated in Figure 10b. Despite the stable decrease in the clamping force, no visual rotational loosening occurred in the loosening test of the multi-bolt structure. These observations are similar to the single-bolt loosening behavior (when obtaining the D-N parameters) and loosening mechanism discussed in our previous study [21].

Figure 9 compares the predicted and test lives of the multi-bolted structure, with black square symbols representing the test lives at the vulnerable points. Consequently, the predicted results are within the ±1.2× error band, demonstrating the good prediction accuracy of this conventional approach for bolt loosening life prediction.

Moreover, bolt loosening was first observed in the more vulnerable regions (test bolt B), as identified by the finite element analysis (FEA) under all test conditions. This further supports the applicability of conventional fatigue life prediction methods for estimating bolt loosening life in structural components. The FEA successfully simulated these vulnerable regions and predicted the loosening life of the complex structures with a high degree of accuracy, showing a strong correlation with the experimental data.

Thus, the study confirms that conventional fatigue life prediction methods are effective for predicting bolt loosening life in bolt-based structures.

## 4. Conclusions

This study proposed a bolt loosening life prediction method by employing material fatigue principles and life prediction techniques under service loading conditions for multi-bolted connections. The following conclusions were drawn:

(1) LDR can be employed to examine bolt loosening life under a two-block loading. The results show that all the loosening test lives for M8 and M6 bolts fall within the ±1.2× error band, which is the accuracy of our method for bolt loosening life calculations.
(2) FEA can be used to identify areas vulnerable to loosening in complex bolted connections. Because the bolt loosening life is significantly dependent on the transverse displacement amplitude, the bolt connection with the largest displacement amplitude is the weakest among the bolted connections of a structure. When multiple loads are applied, various load cases can expectedly find the points of the largest transverse displacement amplitude similar to that in high-cycle fatigue life predictions.
(3) The deformation of bolts in multi-bolted structures is well approximated by the proposed equivalent model. The model, considering both bending and shear deformation, is developed based on Timoshenko beam theory. A comparison with a previously developed FE model incorporating detailed bolt geometry showed a good agreement in hysteresis curves. There are 7.1% and 8.0% differences in stiffness and peak reaction forces, respectively. The structural approximation with computational efficiency can be achieved by the equivalent model.
(4) An experimental setup is configured for the loosening life of multi-bolt structures. The fixture manipulates a displacement amplitude at each bolt connection by providing additional stiffness. Compared with the displacement amplitude at a connection with no additional stiffness, the displacement amplitudes at the other connection, if either of urethane rubber pad, natural rubber pad, or a combination of steel plate/urethan pad (12 t/3 t) are placed, are calculated to be 70%, 96%, 55%, respectively. In the test using the urethan pad, the displacement amplitude at the connection was about 50% of the displacement amplitude at the connection without additional stiffness. The intentional discrepancies of displacement amplitudes are successfully realized through the experimental setup.
(5) The bolt loosening life prediction for the multi-bolt assembly agreed well with the test results. The predicted bolt loosening life was derived by combining the displacement amplitude calculations of the individual bolt with the corresponding D-N curves, and the validation confirmed D (bolt loosening life) = 0.8~1.2. Therefore, our proposed method can predict the bolt loosening life in real structures in a high-cycle fatigue regime (approximately $10^{4}$ cycles).
(6) These findings provide a good example, demonstrating that stress-based life prediction in traditional fatigue analysis is valid for bolt loosening, and we believe that this fundamental principle (e.g., D-N curves, Miner’s rule) can still be applied to other bolt sizes and geometries.

The proposed method provides a practical approach to predicting bolt loosening life. It offers significant potential for industrial applications, particularly in the structural maintenance of bridges, construction equipment, and mechanical assemblies where bolt loosening is a critical concern. Moreover, recently developed self-loosening detection technologies can establish a more reliable maintenance strategy [33,34,35,36].

## Figures and Tables

**Table 1:** Bolt loosening test conditions.

Bolt Size	M8	M6
Initial clamping force	14.3 kN	11 kN
Frequency	10 Hz	
Data sampling rate	1 Hz	

**Table 2:** Bolt loosening life tests under two-block loading.

Test No.	Bolt Size	Two-Block Loading Sequence	Displacement Amplitude
1	M8	Lo-Hi	±0.3 mm, ±0.65 mm
2	M8	Lo-Hi	±0.3 mm, ±0.5 mm
3	M8	Lo-Hi	±0.25 mm, ±0.65 mm
4	M8	Hi-Lo	±0.65 mm, ±0.3 mm
5	M8	Hi-Lo	±0.5 mm, ±0.3 mm
6	M8	Hi-Lo	±0.65 mm, ±0.25 mm
7	M6	Lo-Hi	±0.3 mm, ±0.65 mm
8	M6	Lo-Hi	±0.3 mm, ±0.5 mm
9	M6	Lo-Hi	±0.25 mm, ±0.65 mm
10	M6	Hi-Lo	±0.65 mm, ±0.3 mm
11	M6	Hi-Lo	±0.5 mm, ±0.3 mm
12	M6	Hi-Lo	±0.65 mm, ±0.25 mm

**Table 3:** Stiffness values of the bolt.

Direction	Stiffness (N/mm)
$k_{x}$	12,550
$k_{y}$	12,550
$k_{z}$	502,665

**Table 4:** Bolt loosening life prediction test conditions for multi-bolted structures.

Bolt Size		M8
Initial clamping force		14.3 kN
Displacement	Case 1	±0.3 mm
	Case 2	±0.4 mm
	Case 3	±0.5 mm
Frequency		10 Hz
Data sampling rate		10 Hz

**Table 5:** Measured amplitude displacement of bolted joints for multi-bolted structures.

Input Displacement	Measured Displacement Amplitude
Case 1	Test bolt (A) ±0.1 mm/Test bolt (B) ± 0.3 mm
Case 2	Test bolt (A) ±0.2 mm/Test bolt (B) ± 0.4 mm
Case 3	Test bolt (A) ±0.25 mm/Test bolt (B) ± 0.5 mm

## Data Availability

The original contributions presented in this study are included in the article. Further inquiries can be directed to the corresponding authors.
